# Maxillofacial Trauma in Children

**DOI:** 10.5005/jp-journals-10005-1174

**Published:** 2012-12-05

**Authors:** Chitrita Gupta Mukherjee, Uday Mukherjee

**Affiliations:** Professor, Department of Pedodontia, Buddha Institute of Dental Sciences and Hospital, Gandhi Nagar, Kankarbagh, Patna-800020, Bihar India, e-mail: chitritagm@gmail.com; Professor, Department of Oral and Maxillofacial Surgery, Buddha Institute of Dental Sciences and Hospital, Kankarbagh, Patna, Bihar, India

**Keywords:** Maxillofacial trauma, Children, Facial skeleton, Fracture, Management

## Abstract

Pediatric trauma involving the bones of the face is associated with severe injury and disability. Although much is known about the epidemiology of facial fractures in adults, little is known about injury patterns and outcomes in children. The most common facial fractures were mandible, nasal and maxillary/zygoma. The most common mechanisms of injury are motor vehicle collisions, violence and falls. These fracture patterns and mechanisms of injury varies with age. Cranial and central facial injuries are more common among toddlers and infants, and mandible injuries are more common among adolescents. Although bony craniofacial trauma is relatively uncommon among the pediatric population, it remains a substantial source of mortality, morbidity and hospital admissions. Continued efforts toward injury prevention are warranted. An overview of various types of fractures and their management modalities is discussed, with case reports.

**How to cite this article:** Mukherjee CG, Mukherjee U. Maxillofacial Trauma in Children. Int J Clin Pediatr Dent 2012;5(3):231-236.

## INTRODUCTION

Injuries to the face are far more uncommon than other injuries in children. In addition, pediatric facial injuries are usually minor, such as bruises, hematomas, lacerations or dental trauma. Although trauma is the leading cause of morbidity and mortality in children, and injury remains the most common cause of death in children of 1 year of age and older. Facial trauma associated with severe injury are real challenges to surgeons, and there is subsequent functional and esthetic impact to the growing child and the economic and emotional burden to the patient and family can be overwhelming. Anatomic and developmental differences between pediatric patients and adults alter the diagnosis and management of injury.^1^ This lower incidence of facial fractures partially reflects the underdeveloped facial skeleton and paranasal sinuses of preadolescent children leading to craniofacial disproportion and additional strength of the maxilla and mandible from unerupted dentition.

### Epidemiologic Features

Approximately 5 to 15% of all facial fractures occur in children. The prevalence of pediatric facial fractures is lowest in infants and increases progressively with increasing age. Only 1.0% of facial fractures occur in children younger than 5 years, whereas 1.0 to 14.7% occurs in patients older than 16 years.^2^ Two peaks have been observed in the frequency of such fractures: The first, at the age of 6 to 7 years, is associated with the beginning of school attendance. The second, at 12 to 14 years, is related to increased physical activity and participation in sports during puberty and adolescence.^3^

### Causes of Facial Fracture

Motor vehicle accident is the most common cause (5- 80.2%).^3,4^ It is followed by accidental causes, such as a falls (fall from heights, trees, etc.) (7.8-48%); sports-related injury is the next most common cause (4.4-42%),^3,4^ violence (3.7- 61.1%) and other causes (9.3%).

### Anatomic Distribution of Facial Fractures

Fracture patterns in children are similar to those in adults, but the percentages of fractures found at each anatomic site are different in the pediatric age group. Among pediatric patients, mandibular fractures are by far the most frequent, followed by nasal fractures, orbital, frontal and midfacial fracture.^5^ Complex fractures (nasoorbitoethmoidal) are the least common.

### Effects of Age and Development

In very young children, the frontal protrusion of the cranium and the relative retrusion of the face generate a greater risk of skull fracture than of facial fracture from blunt frontal trauma; the skull absorbs the full force of the initial impact, thus ‘protecting’ the face. With increasing age and physiologic development, the face undergoes a downward and forward projection, with the midface and mandible becoming more prominent. The skull-to-face ratio is 8:1 at birth and 2.5:1 in adulthood: The cranium quadruples in size from birth to adulthood, while the face undergoes a 12-fold increase. Children have a higher resistance to facial fractures and a greater susceptibility to greenstick fractures than adults do, in part because of the structure of bone in the pediatric facial skeleton. The abundance of cartilage and cancellous bone, low mineralization and underdeveloped cortex, along with the more flexible suture lines and indistinct corticomedullary junction, confer greater elasticity and flexibility on the pediatric facial skeleton. The thick layer of adipose tissue that overlies much of the pediatric facial skeleton and the fat pads that surround the upper and lower jaws also help to protect these bones.

### Diagnostic Imaging Methods

To keep the radiation dose as low as reasonably achievable, ultrasonography (USG) may be used instead of radiography for the initial imaging evaluation when the clinical suspicion of fracture is low; if evidence of fracture is found, computed tomography (CT) may be performed for a more detailed evaluation. Regardless of the modality used, a familiarity with the characteristic imaging features of pediatric facial fractures is necessary for accurate image interpretation. In addition, knowledge of the epidemiologic and anatomic distribution of pediatric facial fractures is helpful. The diagnosis of facial fractures in children is difficult, and such fractures are frequently underreported. The interpretation of pediatric facial radiographs is especially challenging and, in many cases, CT is necessary to confirm the diagnosis.

### Surgical Treatment

The rule is simple: It is advisable to be conservative and to prevent growth disturbance, use minimal manipulation. Treatment should be noninvasive whenever possible, and when surgery is necessary, the least invasive procedure and least intrusive devices (e.g. the fewest and smallest plates) should be used. Maxillofacial surgical intervention is indicated only for the repair of severely displaced and comminuted fractures that are likely to cause functional impairment, esthetic deformity or both. Nondisplaced, minimally displaced and greenstick fractures usually are managed conservatively. Internal fixation with semi rigid titanium plates is controversial;^6,7^ moreover, a second surgical intervention is required for removal of the fixation devices.^7,8^

### Mandibular Fractures

Children tend to have only one fracture site, whereas adults usually have more than one site of fracture.^9^ Condylar fractures are often bilateral (20%).^10^ For the evaluation of a suspected mandibular fracture, panoramic radiography is performed.^14^

### Fractures of Frontal Bone and Orbital Roof

Fractures of the frontal bone in young children are common because of the prominence of the forehead, which overhangs the face.

### Fractures of Orbital Floor and Rim

Orbital floor and orbital rim fractures are rare in young children. A common fracture in children at this age is the ‘blowout’ fracture of the orbital floor. The usual mechanism is a direct blow to the eye, with the force of the impact being transmitted downward through the orbital soft tissues to the thin orbital floor. The most characteristic orbital blowout fracture in children, although it is relatively uncommon among pediatric facial fractures, is the so-called trapdoor fracture. This is a greenstick fracture in which a bone fragment protrudes into the sinus while remaining stably attached by a ‘hinge’ of mucoperiosteum to the intact part of the orbital floor, usually on the ethmoidal side. If the displaced fragment springs back into its original position, prolapsed orbital tissues may be entrapped on the maxillary sinus side of the orbital floor. Blowout fractures were treated through a lower eyelid incision with careful release of the entrapped soft tissues and reconstruction of the orbital floor defect. Although autogenous calvarial or split rib bone have been advocated by Zimmermann et al (2005),^1^ alloplastic material with the advantage of avoiding a donor site operation, was used with satisfactory results (Theologie- Lygidakis et al 2007).^13^

### Maxillary and Zygomatic Fractures

Maxillary fractures occur infrequently in the pediatric population: They account for only 1.2 to 20% of pediatric facial fractures, and they virtually do not occur in children younger than 2 years. Their prevalence increases as the maxillary sinuses develop and the permanent teeth erupt, usually around the age of 5 years and it peaks at the age of 13 to 15 years. In children, zygomatic complex fractures often are greenstick fractures involving the lateral wall and floor of the orbit.

## SUMMARY

Isolated facial fractures in children are uncommon overall but occur more frequently in association with major trauma.

Such fractures are found more in boys and their prevalence increases with age. The main causes of pediatric facial fractures are motor vehicle accidents, falls and sports-related injuries. Nasal fractures are by far the most prevalent type of facial fracture among children of all ages, but mandibular fractures are the type of pediatric facial fracture most commonly seen in the hospital setting.^11,12,15^ Closed reduction was selectively applied in condyle fractures and dentoalveolar trauma. The treatment options are open reduction and plate fixation in children. There is no need for wire suspension and only occasional need for IMF. Titanium plates are removed after fracture healing. Fractures of the pediatric facial skeleton have special characteristics, and specific knowledge is necessary for their diagnosis, management and follow-up. To understand the differences between pediatric and adult facial fracture patterns, a familiarity with the processes of facial growth and development is essential because facial fractures in young children have both esthetic and functional repercussions, the early and accurate identification of such fractures is important along with proper management.

### Case 1

A 10-year-old boy sustained periorbital injuries from road traffic accident (RTA), and had left orbital floor fracture. CT confirmed the fracture # and the left orbital floor was explored and repaired with titanium mesh via transconjunctival approach and his pre-operative problems of diplopia and restriction of gaze was resolved (Figs 1A to C).

**Fig. 1A:**
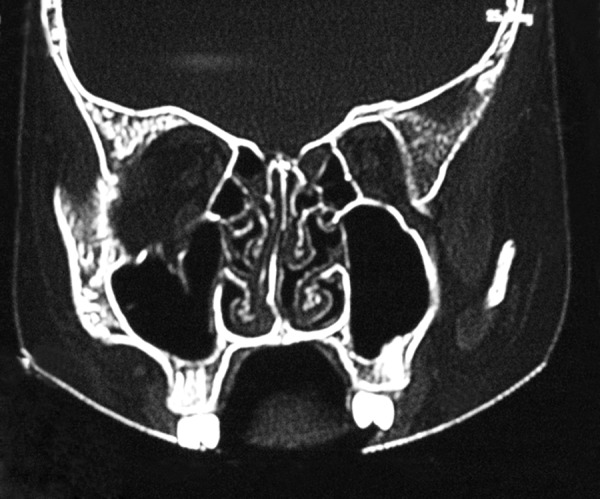
Left floor of orbit fracture in CT scan

**Fig. 1B:**
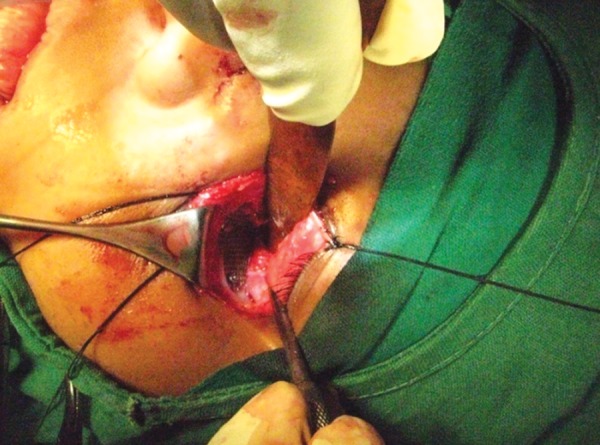
Left floor of orbit fracture, repaired by titanium mesh

**Fig. 1C:**
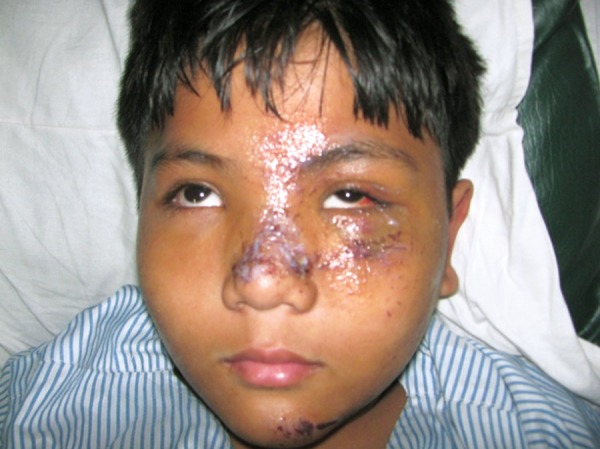
Postoperative picture with normal upward gaze in both right and left eye

### Case 2

A 12-year-old boy sustained a fracture in left parasymphyseal region, which was treated in a different center with reduction followed by circum-mandibular wiring and maxillomandibular fixation. The patient presented 6 months later, with a history of extraoral discharging sinus from the lower border of mandible fractured site, which started after 1 month of the initial operation. The orthopantomogram (OPG) revealed an unerupted left lower 1st premolar which was pushed to the lower border fracture site due to wiring. The area was reoperated with, curettage and removal of the displaced tooth, to restore normal function and occlusion (Figs 2A to D).

**Fig. 2A:**
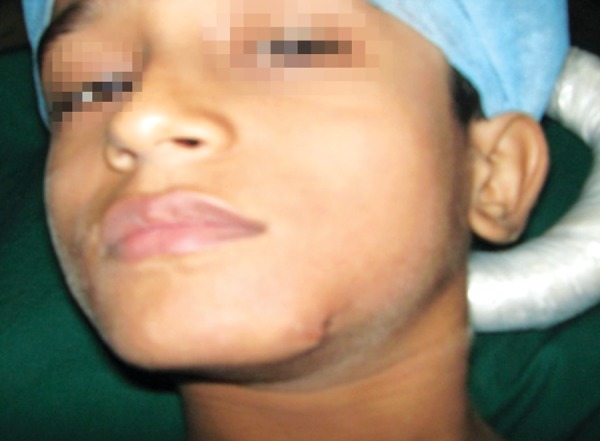
Discharging sinus in an old treated mandible

**Fig. 2B:**
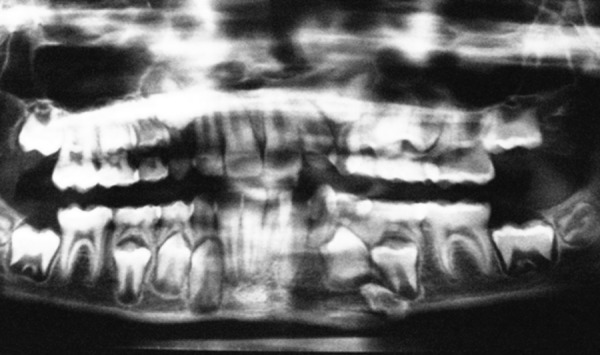
OPG shows left lower 4 pushed to the lower border due to previous circum-mandibular wiring and a persisting infection with sinus

**Fig. 2C:**
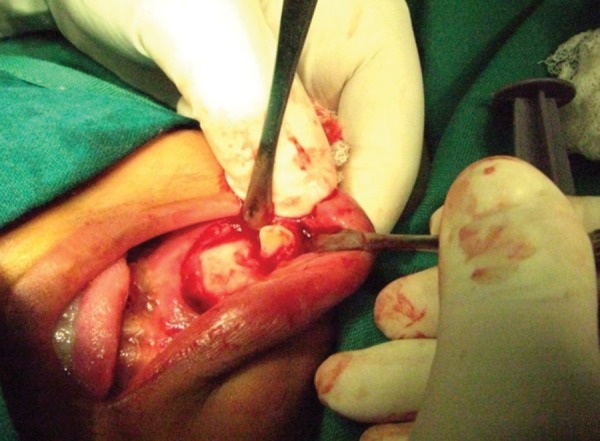
Perioperative picture lower border exposed to elevate malposed tooth and curettage of infection

**Fig. 2D:**
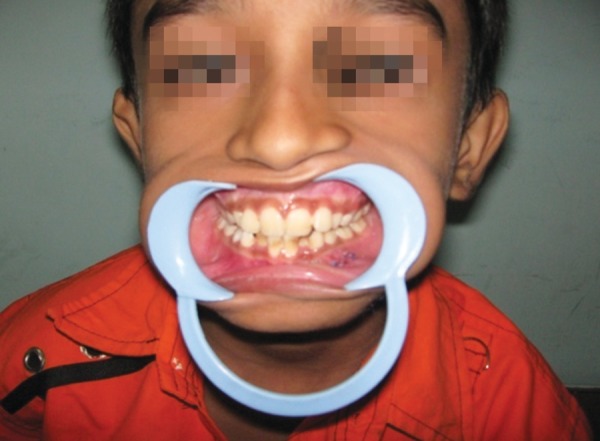
Postoperative occlusion and uneventful healing

### Case 3

A 3-year-old boy sustained a hairline crack in the body of the mandible, following a fall. X-rays revealed an undisplaced hairline crack in the left mandible which was treated conservatively (Figs 3A and B).

**Fig. 3A:**
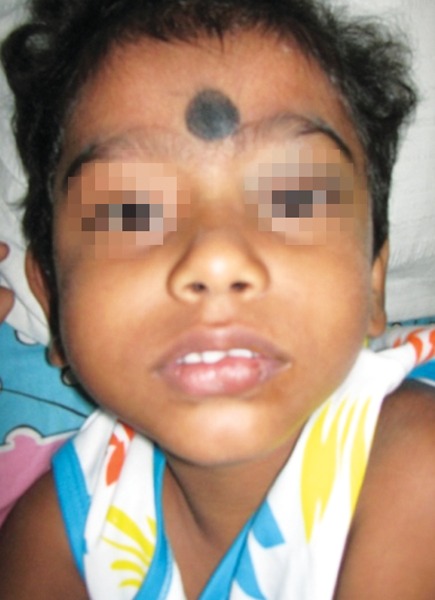
Three-year-old boy history of fall with hairline crack in body of mandible

**Fig. 3B:**
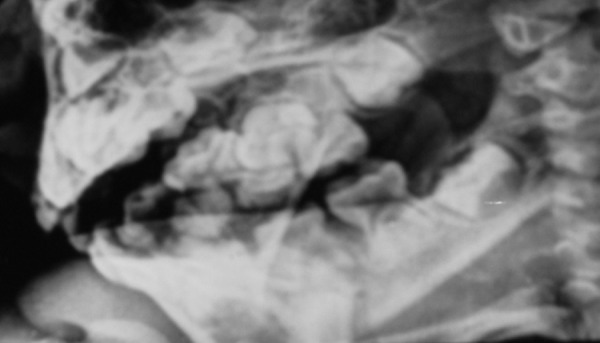
Lateral oblique X-ray of undisplaced body of mandible

### Case 4

A 12-year-old boy sustained an undisplaced fracture in the left parasymphyseal region of mandible, following an RTA, and 3D CT scan revealed the same. The patient was treated conservatively and the patient’s 6-month follow-up pictures reveal normal occlusion and mouth opening (Figs 4A to D).

**Fig. 4A:**
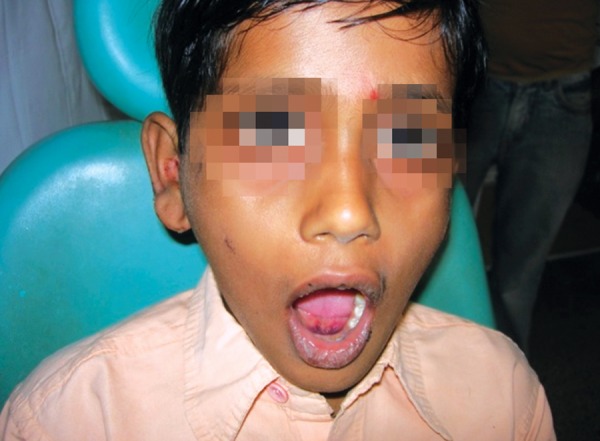
History fall with undisplaced parasymphyseal region

**Fig. 4B:**
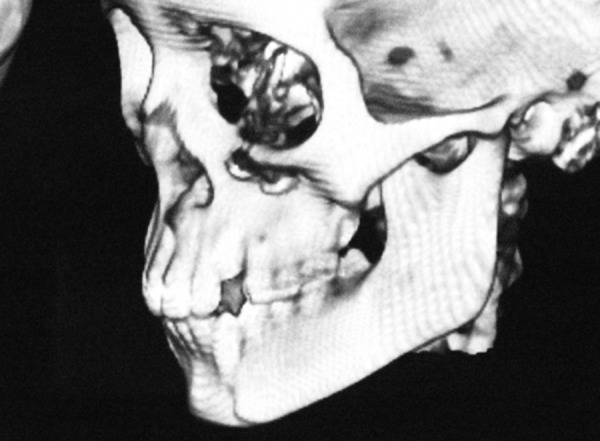
3D CT reformatting

**Fig. 4C:**
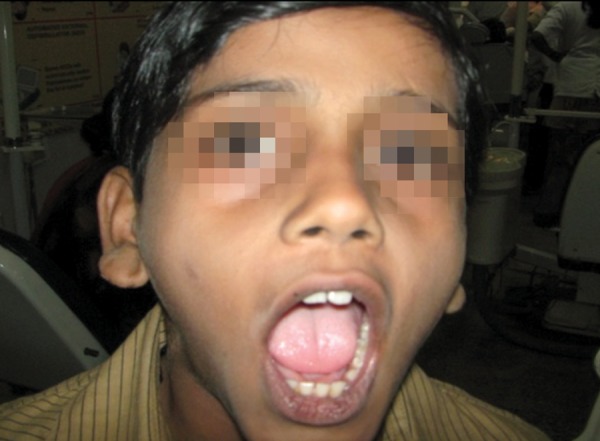
Six month’s follow-up—mouth opening ok

**Fig. 4D:**
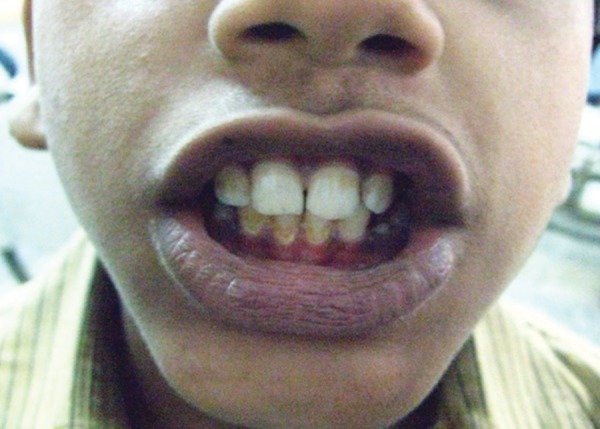
Six months follow-up—occlusion ok

### Case 5

**Figs 5A to C:**
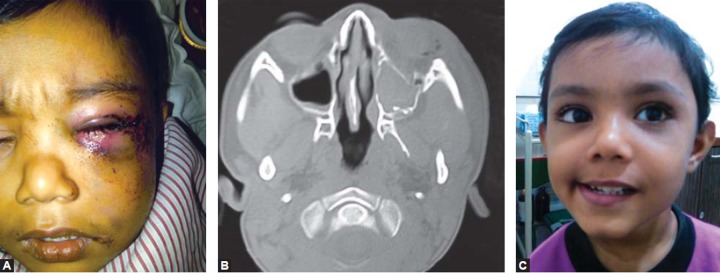
(A) History of fall of 3-year-old, (B) CT scan reveals left anterior wall of maxilla fracture, (C) 5 weeks postoperative

A 3-year-old boy sustained head injury and left periorbital trauma, following a fall from 2 storey open terrace. The CT scan confirmed a left maxillary fracture, which was treated conservatively. The 5 weeks postoperative picture revealed minimal swelling, and normal functionality of mouth opening, occlusion and vision (Figs 5A to C).

### Case 6

An 8-year-old boy sustained left zygomatic-maxilla fracture, following a fall from height (tree) and reported to OPD, 3 weeks later, with a firm left cheek hematoma, X-ray revealed a fractured maxilla, with little displacement. The late presentation made the case a conservative management, with slow and subsequent recovery and function (Figs 6A and B).

### Case 7

A 4-year-old boy sustained head injury with a huge periorbital swelling of right eye, following a fall from height. The 3D CT revealed a fracture nasal bone and frontal bone fracture. The treatment was conservative due to his head injury (Figs 7A and B).

with no obvious deformity

**Fig. 6A:**
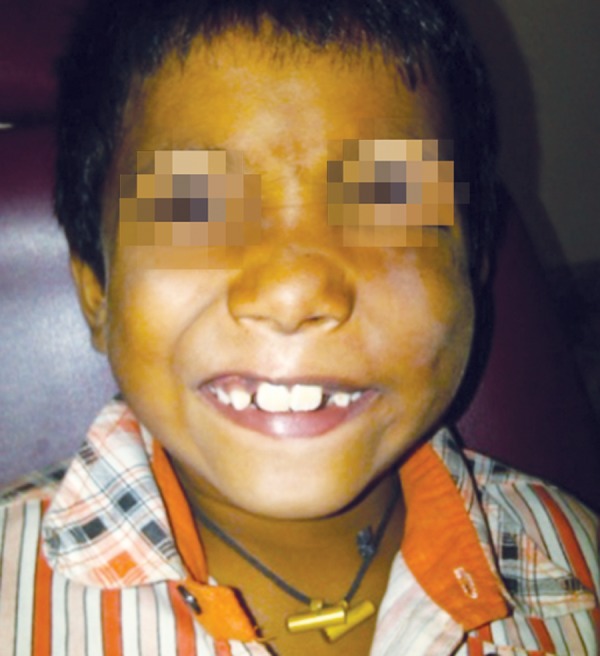
History of fall 8-year-old with left zygomatic-maxilla hematoma and swelling, reported to OPD 3 weeks after fall

**Fig. 6B:**
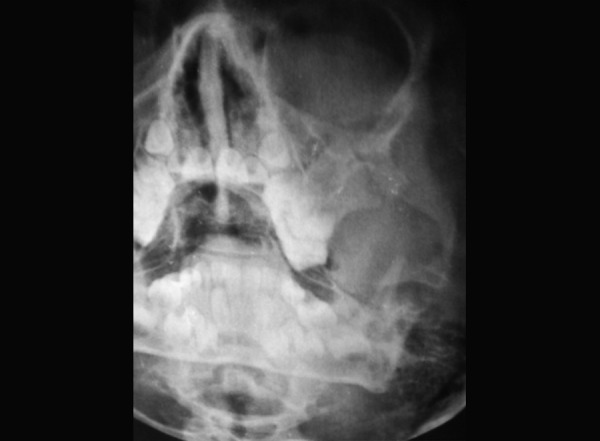
Left maxilla fracture with slight displacement

**Fig. 7A:**
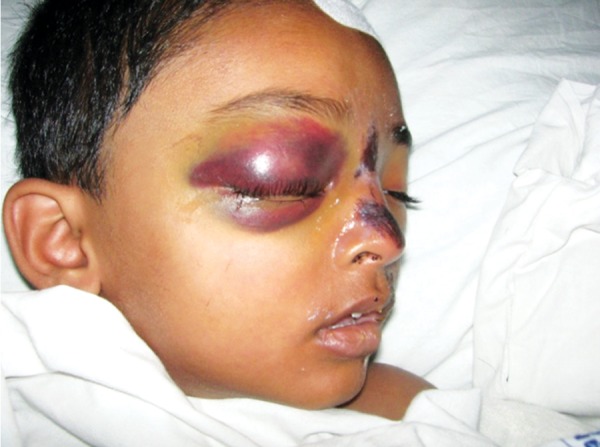
History of fall, with right periorbital swelling and nasal bone fracture

**Fig. 7B:**
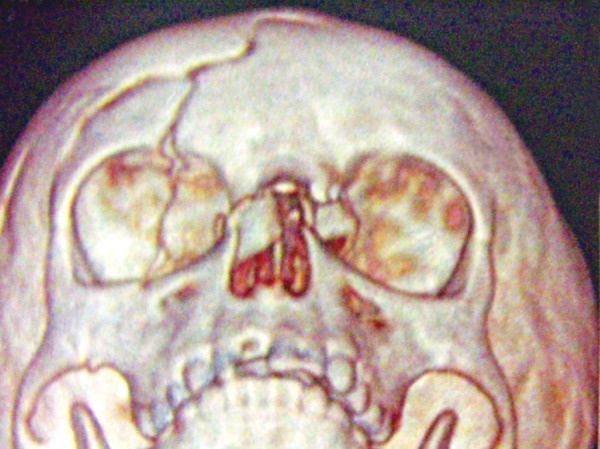
3D CT scan shows fractured right frontal bone and fractured nasal bone
